# Anti-hypertensive Herbs and their Mechanisms of Action: Part I

**DOI:** 10.3389/fphar.2015.00323

**Published:** 2016-01-19

**Authors:** Sara S. Al Disi, M. Akhtar Anwar, Ali H. Eid

**Affiliations:** ^1^Department of Biological and Environmental Sciences, Qatar UniversityDoha, Qatar; ^2^Department of Pharmacology and Toxicology, Faculty of Medicine, American University of BeirutBeirut, Lebanon

**Keywords:** herbal medicine, hypertension, epigenetics, oxidative stress, inflammation, nitric oxide

## Abstract

The use of herbal therapies for treatment and management of cardiovascular diseases (CVDs) is increasing. Plants contain a bounty of phytochemicals that have proven to be protective by reducing the risk of various ailments and diseases. Indeed, accumulating literature provides the scientific evidence and hence *reason d'etre* for the application of herbal therapy in relation to CVDs. Slowly, but absolutely, herbal remedies are being entrenched into evidence-based medical practice. This is partly due to the supporting clinical trials and epidemiological studies. The rationale for this expanding interest and use of plant based treatments being that a significant proportion of hypertensive patients do not respond to Modern therapeutic medication. Other elements to this equation are the cost of medication, side-effects, accessibility, and availability of drugs. Therefore, we believe it is pertinent to review the literature on the beneficial effects of herbs and their isolated compounds as medication for treatment of hypertension, a prevalent risk factor for CVDs. Our search utilized the PubMed and ScienceDirect databases, and the criterion for inclusion was based on the following keywords and phrases: hypertension, high blood pressure, herbal medicine, complementary and alternative medicine (CAM), nitric oxide, vascular smooth muscle cell (VSMC) proliferation, hydrogen sulfide, nuclear factor kappa-B, oxidative stress, and epigenetics/epigenomics. Each of the aforementioned keywords was co-joined with herb in question, and where possible with its constituent molecule(s). In this first of a two-part review, we provide a brief introduction of hypertension, followed by a discussion of the molecular and cellular mechanisms. We then present and discuss the plants that are most commonly used in the treatment and management of hypertension.

## Introduction

Cardiovascular disease (CVD) remains the leading cause of debility and premature death (WHO, 2013), and hence a major public health problem. Out of the major risk factors, which include diabetes, smoking, and dyslipidemia, hypertension is by far the most prevalent trigger for CVDs, and its comorbidity with other risk factors is even more puissant (Yang et al., 2011; WHO, 2013). Hypertension is responsible for around 16.5% of annual deaths worldwide (WHO, 2013), and is indeed the main cause of morbidity and mortality associated with CVDs (Kizhakekuttu and Widlansky, 2010). By 2030, the annual death toll is estimated to reach 23.5 million people (WHO, 2013). In addition to being a major player in the onset of diseases such as atherosclerosis, stroke, peripheral artery disease, heart failure, and coronary artery disease, hypertension can also lead to kidney damage, dementia, or blindness (August, 2004; Freedman and Cohen, 2016). It is important to note that May 17th of every year has been designated World Hypertension Day by the International Society of Hypertension (ISH), and the theme for 2013 World Health Day (7th April) was Hypertension, and hence a focus of considerable attention.

Hypertension is defined as having a systolic blood pressure (SBP) of ≥140 mmHg and a diastolic blood pressure (DBP) of ≥90 mmHg (≥140/≥90 mmHg; Tabassum and Ahmad, 2011). Every 20/10 (SBP/DBP) mmHg increase indicates a higher risk stage of hypertension; stage 1 (140–159/90–99 mmHg), stage 2 (≥160/≥100 mmHg; Archer, 2000; Weber et al., 2014) with the latter stage requiring immediate medical attention (Weber et al., 2014). Importantly, the American Society of Hypertension and ISH recommend that individuals with blood pressure of 120–139/80–89 mmHg be considered as pre-hypertensives (Weber et al., 2014). For targeted therapeutic interest, it is essential to realize that pre-hypertensive individuals are three times more likely to succumb to hypertension at a later stage of life than their normotensive counterparts (Archer, 2000). It is important to note that according to the Eighth Joint National Committee, it is recommended that for the general population, pharmacologic treatment be started at an SBP of 150 mmHg or DBP of 90 mmHg. However, for patients with Chronic Kidney Disease, treatment shall begin when the values of SBP and DBP reach 140 or 90 mmHg or higher, respectively (James et al., 2014).

Elevated blood pressure is categorized into types: primary (essential) and secondary hypertension. Secondary hypertension, which affects 5–10% of hypertensive individuals, is due to identifiable causes, such as diabetes and renal damage, and thus has a relatively higher chance of being treated. On the other hand, essential hypertension is acquired by multiple factors such as diet, age, lifestyle, neurohumoral activity, and interactions (Tabassum and Ahmad, 2011). Since its etiology may be more difficult to ascertain or establish, essential hypertension is more difficult to manage. Interestingly, the percentage of patients with essential hypertension (90–95%) far exceed those with secondary hypertension (Tabassum and Ahmad, 2011).

Many drugs, ranging from diuretics (Indapamide, Furosemide, Amiloride), sympathoplegic agents (clonidine, reserpine), renin inhibitor (Aliskiren), angiotensin converting enzymes (ACE) inhibitors (Enalapril, Captopril, Quinapril), angiotensin receptors blockers (ARBs—Losartan, Irbesartan, Olmesartan), calcium channel blockers (Nifedipine, Verapamil, Diltiazem), α-adrenergic blockers (Prazosin, Doxazosin), β-adrenergic blockers (Nebivolol, Atenolol) to vasodilators (Minoxidil, sodium nitroprusside), are used to manage blood pressure levels in hypertensive patients (Archer, 2000; Susalit et al., 2011). However, a point of interest to physicians and health-care practitioners is the alarming, and rather unfortunate, reality that high blood pressure is managed in only 34% of hypertensive patients (August, 2004; Wang and Xiong, 2012). The major concerns that often delay treatment allude to higher costs of antihypertensive drugs (Susalit et al., 2011), their availability and accessibility (Wang and Xiong, 2012), the undesired side effects of antihypertensive drugs (Susalit et al., 2011; Wang and Xiong, 2012) and the reduced patient compliance to consume more than a pill per day (August, 2004). Taking this into account, hypertensive patients, especially those dwelling in rural areas, seek alternative approaches such as herbal remedies for their treatment of hypertension and other diseases.

## Herbal remedies

The use of herbal medicine as a treatment modality has significantly increased over the last decade (Frishman et al., 2009). This is due to several factors, principal of which is that herbal medicine is a cheaper alternative with fewer undesired side effects (Frishman et al., 2009; Susalit et al., 2011; Tabassum and Ahmad, 2011). However, the increased desire to use herbal treatment is not a reflection of the economic status of an individual from a certain region or a country. Indeed, 70% of the population in developed nations have resorted to Complementary and Alternative Medicine (CAM) for treatment purposes, and herbal medicine forms a large proportion of its application (WHO, 2008). Further, the usage of CAM in developing countries is becoming even more pronounced (WHO, 2008). Evidently, the rationale for the use of herbal and plant remedies is definitely not surprising, considering the fact that they contain thousands of bioactive components that have known therapeutic applications (Pan et al., 2013). Indeed, plants and herbs have actually provided a starting point for synthesis of over 50% of currently used pharmaceutical drugs (Pan et al., 2013). The pharmacopeia includes ephedrine (from *Ephedra sinica*), aspirin (from *Salix alba*), lovastatin (from *Monascus purpureus*), reserpine (from *Rauwolfia serpentina*), and taxol (from *Taxus brevifolia*; Frishman et al., 2009). Remarkably, reserpine (which depletes adrenergic neurotransmitters) still remains an effective treatment for hypertension (Weber et al., 2014).

Importance of plants and herbs, *per se*, in the medical field must not be overlooked as they have been used throughout human history. Herbal plant-based formulations or drugs are pivotal to Traditional practices in Chinese, Ayurvedic, and Unani Tibb medicine, which is practiced worldwide. Overall, this may explain the increasing interest in panning out the beneficial health effects of various plants and herbs in different diseases including hypertension (Tabassum and Ahmad, 2011). In this review, we focus to provide a summary of different plants that have been reported to exhibit antihypertensive properties, and that can specifically mitigate anti-inflammatory causes in arterial hypertension. Moreover, where information is available, we have discussed botanical-induced improvements in renal biology.

## Molecular pathogenesis of hypertension

Hypertension is characterized by arterial derangement in the vascular tree, affecting large conduit arteries (such as aorta), small resistance size arteries (150–400 μm), and the microcirculation (arterioles and capillaries). Increased arterial reactivity (sensitivity and potency) due to dysregulation in endothelial nitric oxide synthase (eNOS) and pro-oxidant enzymes, enhanced basal and activated calcium levels due to overactive transmembrane calcium permeability through calcium channels, and/or coexistence of vascular smooth muscle cell (VSMC) hyperplasia and hypertrophy (vascular remodeling) can all lead to increased vasoconstriction. These pathological events lead to an increased ratio of vessel wall thickness as compared to the dimensions of the arterial lumen (Folkow, 1990). It is this increased ratio that plays a major role in precipitating hypertension. Below we discuss some of the major mechanisms implicated in the pathogenesis of hypertension. Then we discuss the most commonly used herbs that ameliorate blood pressure by modulating these mechanisms.

### Vascular smooth muscle cell (VSMC) proliferation

**Figure 1:**
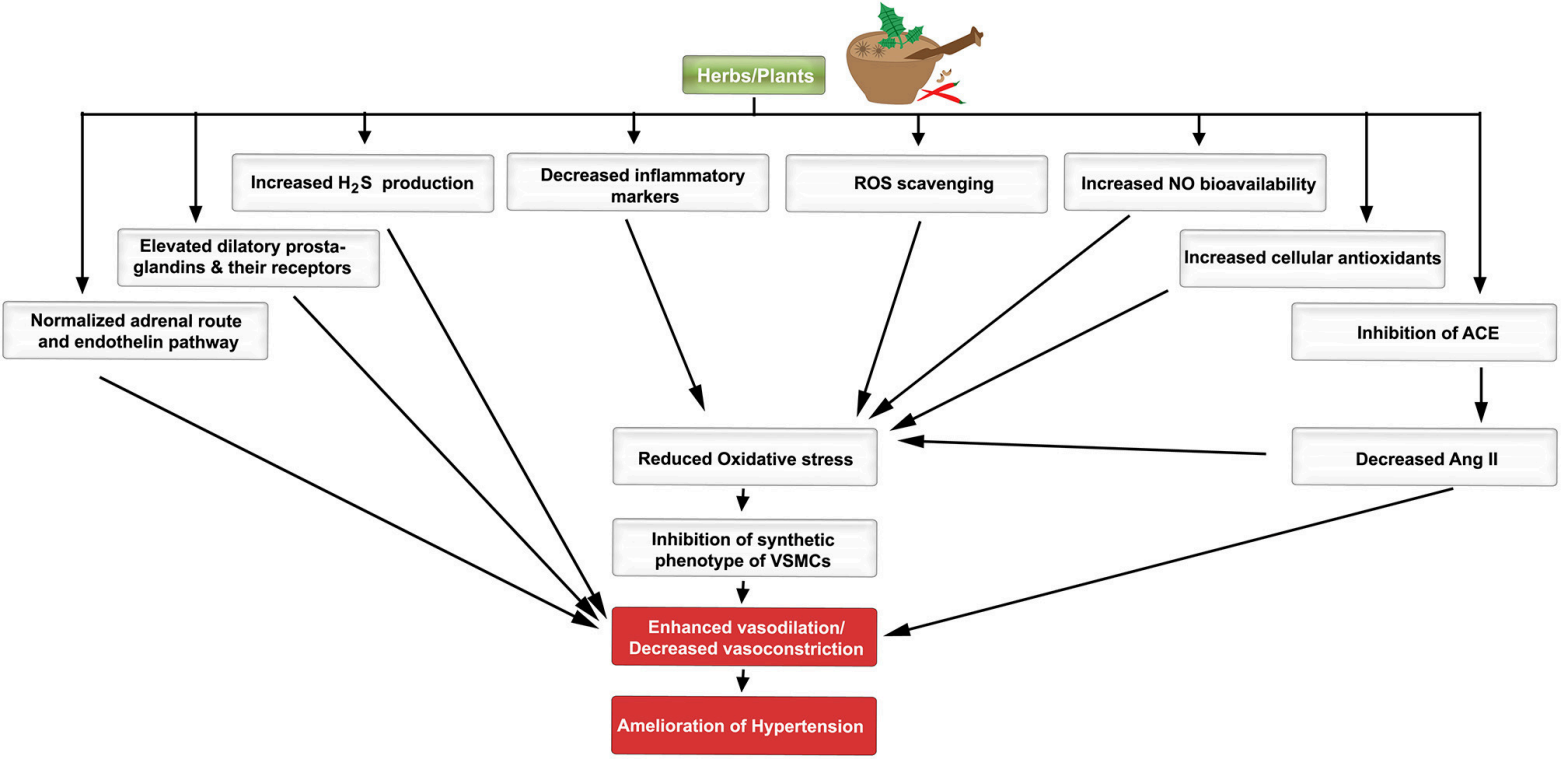
**A schematic diagram indicating the favorable effects of plants/herbs on the molecular pathogenesis of hypertension**. Different molecular, biochemical, and cellular pathways are favorably modulated by herbs/plants or their extracts.

VSMCs participate in the pathogenesis of hypertension (Oparil et al., 2003; Lacolley et al., 2012), and their proliferation contributes to increased peripheral resistance by decreasing arterial diameters (Oparil et al., 2003; Figure 1). For an understanding of these intricate alterations, it is essential to examine the modulating factors that stimulate or inhibit VSMC growth for the treatment of hypertension. Growth factors impel the cell into entering cell cycle until the G1 phase, the first check point (Marx et al., 2011). These growth factors include: platelet-derived growth factor (PDGF; Itoh et al., 1993; Rudijanto, 2007; Marx et al., 2011), fibroblast growth factor (FGF; Itoh et al., 1993; Rudijanto, 2007; Marx et al., 2011) endothelin-1, thrombin, interleukin-1 (IL-1; Rudijanto, 2007), and visfatin (Miao and Li, 2012). Angiotensin II (Ang II) can also promote cell cycle progression especially that it can regulate the expression of both basic FGF (Itoh et al., 1993) and epidermal growth factor receptor (EGFR; Inagami and Eguchi, 2000).

Amongst the several VSMC growth inhibitors are nitric oxide (NO), cyclic guanosine monophosphate (cGMP; Pilz and Casteel, 2003), transforming growth factor-beta (TGFβ; Itoh et al., 1993; Rudijanto, 2007), and adenosine monophosphate-activated protein kinase (AMPK; Song and Zou, 2012). For example, 5-aminoimidazole-4-carboxamide ribonucleotide (AICAR), a membrane-permeable activator of AMPK, inhibits cell cycle progression, and migration of VSMCs as well as vascular remodeling following injury (Stone et al., 2013). Moreover, rosiglitazone, a peroxisome proliferator-activated receptor gamma (PPARγ) agonist, acts via protein kinase G (PKG) to exert an anti-proliferative effect on VSMCs in models of angioplasty-induced vascular injury (Yang et al., 2013). Contextually, complex regulatory interactions exist between growth factors and the VSMC phenotype (Itoh et al., 1993). The balance between the pro-proliferative and anti-proliferative signals determines, to a large extent, the phenotype of VSMCs. As will be discussed later in this review, many plants and herbs indeed ameliorate increased blood pressure by favorably modulating the VSMC phenotype.

### Endothelial cells

The endothelial cell layer is no longer considered as an inert entity. It rather plays significant roles in many aspects of homeostasis along the cardiovascular network. Endothelial function is regulated and maintained by a variety of cell surface receptors, some of which induce the release of vasoactive substances to regulate vascular tone and smooth muscle cell proliferation (Drexler and Hornig, 1999). Local and circulating cues stimulate the vascular endothelium to release vasodilators (NO, prostacyclin, and endothelial-derived hyperpolarizing factor) and vasoconstrictors (endothelin, thromboxane, and PDGF; Iglarz and Clozel, 2007). When there is an imbalance between these vasoactive agents, an increased production of reactive oxygen species (ROS) may result, which then leads to endothelial dysfunction and eventually hypertension (Virdis and Taddei, 2011; Montezano and Touyz, 2012; Silva et al., 2012; Figure 1).

Endothelial dysfunction anticipates the progression of anatomically overt vascular disease, which robustly correlates with hypertension. However, endothelial derangement could be reversed by modification therapy, such as with herbal remedies. This fact will be highlighted in different sections of this review.

### Repertoire of signaling molecules

Assuming a homeostatic imbalance, the following signaling molecular entities become integral to the pathogenesis of hypertension (Ong and Whitworth, 2011; Montezano and Touyz, 2012). Fortunately, a diverse range of plant and herbal extracts and their individual metabolites can modulate signaling cascades implicated in the physiology of the cardiovascular system (see Section Herbs and Spices Most Commonly Used for Treatment of Hypertension, below). These herbs are not only vasculo-protective but they could also potentially reverse the changes in hypertension. This particularly applies if the alterations in hypertensive patients are addressed prior to reaching a decompensated state.

#### Reactive oxygen species

In good health, the activity of pro-oxidants is balanced by anti-oxidative agents. However, when this equilibrium is disturbed, the un-orchestrated milieu leads to pathologic states, such as hypertension, atherosclerosis, and other vascular complications (Figure 1; Montezano and Touyz, 2014). ROS such as superoxide anions (${\text{O}}_{2}^{∙-}$) and hydroxyl ions (OH^−^) play a major role by promoting an environment of oxidative stress, which is a primary cause in the pathogenesis of hypertension (Zeng et al., 2009; Song and Zou, 2012; Montezano et al., 2015). ROS are produced from the reactivity of nicotinamide-adenine dinucleotide phosphate (NADPH) oxidase and many other enzymatic reactions particularly ones related to the electron transport chain within the mitochondria (Zeng et al., 2009; Dharmashankar and Widlansky, 2010; Drummond et al., 2011; Song and Zou, 2012). The oxidant molecules are inactivated by antioxidant enzymes such as superoxide dismutase (SOD), catalase (CAT), glutathione peroxidase (GPX), and non-enzymatic substances like reduced glutathione (GSH; Song and Zou, 2012). Recent evidence suggests that AMPK can also suppress the activity of NADPH oxidase (Song and Zou, 2012). An increase in the amount of ROS, and the corresponding fall in intracellular endothelial antioxidant levels (Zeng et al., 2009; Drummond et al., 2011; Song and Zou, 2012), signals an oxidative stress state. The oxidative stress leads to endothelial cell dysfunction and vascular smooth muscle remodeling by reducing NO bioavailability (Zeng et al., 2009; Drummond et al., 2011). ROS are also responsible for the oxidation of low-density lipoprotein (LDL), which results in inflammation and increased proliferation of VSMCs (Slevin et al., 2012). Both inflammation and enhanced proliferation significantly trigger plaque formation (Slevin et al., 2012) which in turn may contribute to increased blood pressure. Interestingly, blocking ROS production by exposure to antioxidants has been shown to reduce blood pressure in rodents (Zeng et al., 2009).

A key molecule that regulates oxidative stress in VSMCs is the transcription factor erythroid 2-related factor 2 (Nrf2, or nuclear factor erythroid 2-like 2). Under physiological conditions, Nrf2 activity is inhibited by its endogenous inhibitor Kelch-like ECH-associated inhibitor 1 (Keap1; Villeneuve et al., 2013). On the other hand, when oxidative stress ensues, Keap1 releases Nrf2, which then translocates to the nucleus and promotes expression of different antioxidant enzymes such as CAT, GPX, heme oxygenase-1 (HO-1), SOD (Kern et al., 2007). Pertinently, various phytochemicals suppress oxidative stress by upregulating antioxidant enzymes through the Keap1–Nrf2 pathway (Tao et al., 2013; Niture et al., 2014).

#### Nitric oxide

Nitric oxide (NO) is often recognized as an important indicator of vascular health. It plays an important role in blood pressure regulation due to its vasodilating potency (Francis et al., 2010) as well as its ability to inhibit aggregation of platelets and proliferation of VSMCs (Zeng et al., 2009). NO is produced from L-arginine (Francis et al., 2010) by NO synthases (NOS) such as endothelial NOS (eNOS; Francis et al., 2010). After its release, NO translocates into VSMCs and activates soluble guanylate cyclase (sGC), which then catalyzes the conversion and cyclization of guanosine triphosphate (GTP) to cGMP (Francis et al., 2010). cGMP then binds to cGMP-dependent protein kinases (PKGs; Francis et al., 2010) which modulate calcium levels and the consequent contraction of VSMCs (Francis et al., 2010). Within seconds of its production, NO reacts with superoxide anions (Drummond et al., 2011) to produce peroxynitrite (ONOO^−^; Drummond et al., 2011). This in itself is a selective oxidant and can precipitate oxidative stress as well as diminish the vasodilatory effect of NO (Drummond et al., 2011). As such, the concentration of ROS has a direct impact on the bioavailability of NO. Interestingly, oxidative stress can also induce eNOS uncoupling, so that eNOS generates free radicals instead of NO. For example, stimulation of NADPH oxidase leads to the generation of superoxide anion (${\text{O}}_{2}^{∙-}$), which reacts with NO to form the potent oxidizing agent, peroxynitrite. This peroxynitrite then oxidatively degrades the eNOS cofactor tetrahydrobiopterin (BH_4_) to the inactive dihydrobiopterin (BH_2_). Effectively, this yields more superoxide anion that eventually depletes BH_4_. Therefore, a shift in balance from NO to superoxide formation ensues; this is referred to as uncoupling of NOS (Michel and Vanhoutte, 2010). As such, the interplay between NO and the regulatory mechanisms governing its bioavailability are thus intimately intertwined with vascular tone and endothelial dysfunction (Montezano and Touyz, 2012).

#### Hydrogen sulfide

Hydrogen sulfide (H_2_S) is an important biological mediator produced in VSMCs and potentially endothelial cells via the catalysis of L-cysteine by the enzyme cystathionine γ-lyase (CSE; Calvert et al., 2010; Pan et al., 2012). It has been reported that deficiency in H_2_S production is positively correlated with pathophysiology of hypertension in several animal models (Benavides et al., 2007; Liu et al., 2012). Indeed, CSE activity and H_2_S levels were notably decreased in the aorta and plasma of SHR, respectively (Yan et al., 2004). In addition, there was a significant increase in BP levels of Wistar Kyoto (WKY) rats treated with DL-propargylglycine (PPG), a CSE inhibitor (Yan et al., 2004). Furthermore, administration of H_2_S is sufficient to abolish the N^ω^-nitro-L-arginine methyl ester hydrochloride (L-NAME, an inhibitor of nitric oxide synthase)-induced hypertension in WKY rats (Zhong et al., 2003).

At the molecular and cellular levels, H_2_S exhibits a vasorelaxant effect on VSMCs by increasing intracellular levels of cGMP (Bucci et al., 2012) and opening ATP-dependent potassium channels (K_ATP_; Banerjee et al., 2002; Calvert et al., 2010; Bucci et al., 2012; Pan et al., 2012). Interestingly, H_2_S has also been reported to prevent vascular inflammation (Calvert et al., 2010; Pan et al., 2012), reduce ROS production, depress Ang II and ACE levels, potentiate antioxidant mechanisms, diminish VSMC proliferation, and induce NO synthesis (Benavides et al., 2007).

#### Angiotensin II

The Renin–Angiotensin–Aldosterone (RAA) system plays a pivotal role in regulating blood pressure (Nguyen Dinh Cat and Touyz, 2011). Activation of the RAA system in response to a decrease in cardiac output leads to the secretion of renin, which in turn catalyzes the conversion of angiotensinogen to angiotensin I (Ang I). Ang I is then cleaved by Angiotensin Converting Enzyme (ACE)-1 (ACE-1) to form Angiotensin II (Ang II). Ang II-mediated hypertension occurs by promotion of sodium and water retention as well as enhancement of vasoconstriction by binding to the angiotensin type 1 (AT_1_) receptor (Morgan, 2003; Nguyen Dinh Cat and Touyz, 2011; Savoia et al., 2011). Activation of AT_1_ receptors is known to trigger the proliferation of VSMCs (Castro et al., 2010; discussed earlier). The fact that ACE is present in several tissues, including arteries, indicates that Ang II can be formed locally within the arteries themselves. Indeed, there is a higher concentration of Ang II in the vasculature of hypertensive compared to normotensive rats (Ma et al., 2010). Taken together, these observations may explain why inhibiting ACE is an attractive approach for antihypertensive therapies (Morgan, 2003; Bernstein et al., 2013). Ang II induces aldosterone production (Oparil et al., 2003; Manrique et al., 2009; Ma et al., 2010) and cardiac muscle cell growth (Manrique et al., 2009; Ma et al., 2010; Song and Zou, 2012). Blockade of AT_1_ receptors improves endothelial dysfunction (Virdis et al., 2011) and has been reported to decrease BP (Kang et al., 2009). Accumulating evidence demonstrates that Ang II also stimulates NADPH oxidase to generate ROS (Ma et al., 2010; Virdis et al., 2011). In addition, Ang II may increase the sympathetic nervous system (SNS) activity (Manrique et al., 2009), which participates in pathogenesis of hypertension by elevating cardiac output and increasing peripheral vascular resistance (Oparil et al., 2003).

#### Nuclear factor kappa B

Inflammation is well-documented to contribute to vascular remodeling and consequent hypertension (Vazquez-Prieto et al., 2011). Nuclear Factor kappa B (NF-κB), a transcription factor, is known to partake in the pathology of hypertension. It induces endothelial cell dysfunction, oxidative stress, and inflammation (Li and Zhuo, 2008; Kang et al., 2009) through the release of pro-inflammatory cytokines, such as tumor necrosis factor-alpha (TNF-α) and interleukin-6 (IL-6; Kang et al., 2009).

It has been shown that increased expression and activation of NF-κB contributes to renal injury and hypertension (Elks et al., 2009). Indeed, suppressing NF-κB with pyrrolidine dithiocarbamate (PDTC) decreased SBP in spontaneous hypertensive rats (SHRs; Elks et al., 2009). Interestingly, PDTC also suppressed the higher concentration of cytosolic and mitochondrial ROS in kidneys of these SHRs (Elks et al., 2009).

Activation of NF-κB could be induced by several factors such as Ang II (Kang et al., 2009), ROS (Kang et al., 2009), and/or tumor necrosis factor-alpha (TNF-α; Mathew and Biju, 2008; Zhang et al., 2009; Vazquez-Prieto et al., 2011). Interestingly, TNF-α-induced ROS production, which drastically impacts endothelial dysfunction, is shown to be mediated by NF-κB (Zhang et al., 2009). Moreover, NF-κB plays an important role in attenuation of ANG II-induced pressor response (Kang et al., 2009), regulation of AT_1_ receptors (Bhatt et al., 2014; Luo et al., 2015), as well as induction of oxidative stress (Mathew and Biju, 2008; Zhang et al., 2009; Vazquez-Prieto et al., 2011). Further, NF-κB can increase proliferation, decrease apoptosis of endothelial cells, as well as stimulate expression of vascular cell adhesion molecule-1 (VCAM-1; Vazquez-Prieto et al., 2011; Jiang et al., 2013b). Taken together, these NF-κB-induced changes can lead to the derangement of endothelial function and vascular tone.

## Herbs and spices most commonly used for treatment of hypertension

Secondary metabolites of herbs and spices exhibit anti-hypertensive effects. Here, we present a comprehensive alphabetical list of herbs for which sound evidence suggests they could be beneficial in hypertension therapy (Tables 1–**6**; Figure 1).

**Table 1:** **Commonly used antihypertensive plants with antioxidant activity**.

**Herb**	**Effect**	**Concentration/Dose**	**Experimental setting/Model**	**References**
*Allium sativum*	Scavenges ROS	3 mg/ml	Human neutrophils	Morihara et al., 2011
	Increases antioxidants	500 mg/ml	2K-1C rats	Drobiova et al., 2011
		125–2000 mg/kg	Wistar albino rats' hearts	Banerjee et al., 2002
	Reduces NADPH activity	150 and 400 mg/kg	Fructose-fed rats	Vazquez-Prieto et al., 2011
*Andrographis paniculata*	Scavenges ROS	0.7–2.8 g/kg	SHR	Zhang and Tan, 1996
*Apium graveolens*	Increases antioxidants	1 ml/kg (of different extracts)	CCl_4_-treated mice	Popovic et al., 2006
*Camellia sinensis*	Scavenges ROS	1–5 μg/ml	Superoxide-generating system	Nakagawa and Yokozawa, 2002
	Decreases NADPH oxidase	13.3 g/L	STZ fed SHR	Ribaldo et al., 2009
	Increases antioxidants	0.1%	Streptozotocin (STZ)-fed Sprague-Dawley rats	Thomson et al., 2012
		1% Green Tea Extract	C57BL/6 mice	Newsome et al., 2014
	Inhibits eNOS uncoupling	5 g/kg	STZ fed SHR	Faria et al., 2012
*Coptis chinensis*	Increases antioxidants	150 mg/kg	Atherosclerotic renovascular disease (ARD) Wistar rats	Wan et al., 2013
	Decreases NADPH oxidase	150 mg/kg	ARD Wistar rats	Wan et al., 2013
*Coriandrum sativum*	Inactivates ROS produced by β-adrenoceptor stimulation	200 and 300 mg/kg	Isoproterenol-induced cardiotoxicity in male Wistar rats.	Patel et al., 2012
	Increases antioxidants	200 mg/kg	CCl_4_-induced hepatotoxicity in Wistar albino rats	Sreelatha et al., 2009
*Crataegus* spp.	Scavenges ROS	100–400 μg/ml	enzymatic assay	Cheng et al., 2013
*Crocus sativus*	Reduces oxidative stress	200 mg/kg	BeCl_2_-treated Wistar rats	El-Beshbishy et al., 2012
	Increases antioxidants	200 mg/kg	BeCl_2_-treated Wistar rats	El-Beshbishy et al., 2012
		20–80 mg/kg	Genotoxins-treated Swiss albino mice	Premkumar et al., 2003
*Hibiscus sabdariffa*	Scavenges ROS	2 mg/ml	CCl_4_-induced hepatotoxicity in rat liver	Ajiboye et al., 2011
	Increases antioxidants	10 g extract (powder), dissolved in 200 mL water	Healthy humans	Frank et al., 2012
*Panax*	Increases antioxidants	60–120 μM	Hypoxia/Reoxygenation-induced oxidative injury in rat cardiomyocytes	Doh et al., 2013
*Salviae miltiorrhizae*	Reduces ROS	100 μg/ml	Sprague-Dawley rat thoracic aortic VSMCs	Cho et al., 2013
	Increases antioxidants	5 g extract/time, twice per day; 60 days	Chronic heart disease (CHD) patients	Qian et al., 2012
*Zingiber officinale*	Scavenges ROS	0–60 μM	Enzymatic assay	Shin et al., 2005
	Inhibits lipid peroxidation	0.05 mg/ml	Rat heart	Akinyemi et al., 2013

### *Allium sativum* (garlic)

Garlic's multi-fold therapeutic effects have been recognized for thousands of years amongst different cultures around the world, and continues to attract interest from pharmacologists and health practitioners (Frishman et al., 2009; Qidwai and Ashfaq, 2013; Table 2). This herb is not only known for its hypotensive capacity, but is also characterized by anti-inflammatory, antioxidant, antibacterial, hypocholesteremic, and anti-cancer properties (Banerjee et al., 2002; Mousa and Mousa, 2007; Frishman et al., 2009; Qidwai and Ashfaq, 2013). For health benefits, garlic can be consumed in different forms, such as raw, aged, an aqueous extract, oil, and in powder form (Banerjee et al., 2002; Frishman et al., 2009; Ried et al., 2013).

**Table 2:** **Commonly used antihypertensive plants with vasorelaxant activity**.

**Herb**	**Effect**	**Concentration/Dose**	**Experimental setting/Model**	**References**
*Allium sativum*	Increases NO	Reported only as garlic extract	Human umbilical vein endothelial cells	Mousa and Mousa, 2007
		0.8 mg/ml	Rat isolated pulmonary arteries	Ku et al., 2002
	Increases eNOS	150 and 400 mg/kg/day	Fructose-fed Wistar rats	Vazquez-Prieto et al., 2011
	Increases H_2_S	500 μg/ml	Sprague-Dawley rat aortic rings	Benavides et al., 2007
	Inhibits ACE		Fructose-fed rats	Sendl et al., 1992
*Andrographis paniculata*	Increases NO	1 mg/ml	Isolated hearts from Sprague-Dawley rats	Awang et al., 2012
	Blocks Ca^2+^ channels	1 mg/ml	Isolated hearts from Sprague-Dawley rats	Awang et al., 2012
	Reduces ACE	0.7–2.8 g/kg	SHR	Zhang and Tan, 1996
*Apium graveolens*	Blocks Ca^2+^ influx	48 mM	Rat isolated aortic rings	Ko et al., 1991
*Bidens pilosa L*.	Ca^2+^ antagonists	0.32 mg/ml	KCl-treated rat aorta	Nguelefack et al., 2005
	Mechanism not determined	40 mg/ml	High-fructose fed Wistar rats	Dimo et al., 2002
*Camellia sinensis*	Increases flow-mediated dilation (FMD)	2 g in 250 ml boiled water/day	Brachial arteries of subjects with elevated cholesterol level	Hodgson et al., 2002
		450 and 900 mL	Brachial arteries of coronary heart disease patients	Duffy et al., 2001
	Increases NO	580 mg	Healthy male smokers (preclinical pilot)	Oyama et al., 2010
	Inhibits eNOS uncoupling	5 g/kg daily	Diabetic SHR	Faria et al., 2012
	Blocks AT_1_ receptor	0.1%	STZ-fed Sprague-Dawley rats	Thomson et al., 2012
*Coptis chinensis*	Upregulates eNOS expression	2.99, 3.45, 5.81, and 6.14 g/L	Rat isolated cardiomyocytes (insulin-induced hypertrophy)	Zhang et al., 2011
		2.99, 3.45, 5.81, and 6.14 g/L	Isolated thoracic aorta rings from CIHH rats	Zhang et al., 2011
	Decreases EMP	1.2 g/L	Healthy humans	Affuso et al., 2010
	Blocks Ca^2+^ channels	5.18 and 6.14 g/L	Isolated thoracic aorta rings from CIHH rats	Zhang et al., 2011
*Crataegus* spp.	Activates eNOS	100 mg/kg/day	L-NAME-induced hypertensive rats	Koçyildiz et al., 2006
		100 μg	Male Wistar Rat isolated aortic rings	Brixius et al., 2006
		100 μg	Human isolated mammarian arterial rings	Brixius et al., 2006
*Crocus sativus*	Activates eNOS	0.1–0.5 ml/kg	ischemia-reperfusion (IR) in rats	Bharti et al., 2012
	Blocks Ca^2+^ channels	1 and 5 mg%	Guinea pig Isolated heart	Boskabady et al., 2008
*Cymbopogon citratus*	Increases NO bioavailability	30 mg/ml	Isolated aorta from SHR	Devi et al., 2012
		30 mg/ml	Isolated aorta from WKR	Devi et al., 2012
		1–20 mg/kg	Rat isolated thoracic aorta	Bastos et al., 2010
	Inhibits Ca^2+^-influx	1–20 mg/kg	Rat isolated thoracic aorta	Bastos et al., 2010
*Hibiscus sabdariffa*	Increases NO	0.3 mg/ml	SHR isolated aorta	Ajay et al., 2007
		1500–2500 mg/kg	Not clear	Alarcon-Alonso et al., 2012
	Blocks Ca^2+^ channels	10 ng^−1^ mg/ml	SHR isolated aorta	Ajay et al., 2007
	Opens K_ATP_ channels	10^−4^–10^−1^ g/L	Male Wistar rat thoracic aorta	Sarr et al., 2009
	Reduces ACE	250 mg	Stage 1 and 2 hypertensive humans	Herrera-Arellano et al., 2007
*Nigella sativa*	Blocks Ca^2+^ channels	2–14 mg/ml	Rat isolated aorta	Niazmand et al., 2014
*Panax*	Increases eNOS	150 μg/ml	SHR adrenal medulla	Jang et al., 2011
*Salviae miltiorrhizae*	Increases NO	0–10 mg/ml (of SalB, a major ingredient of this plant)	Rabbit thoracic aortic rings	Shou et al., 2012
	Opens K_ATP_ channels	0.25–2 mg/ml	SHR aorta	Ng et al., 2011
	Blocks Ca^2+^ channels	300–1000 μg/ml	Porcine coronary rings	Hu et al., 2012
		10.39 ± 1.69 μM	Rat coronary arterial rings	Lam et al., 2008
	Reduces ACE activity	0.05 mg/ml	Rat heart	Akinyemi et al., 2013

Several mechanisms have been alluded to in the explanation of garlic's hypotensive effects (Shouk et al., 2014). These are based on garlic's organo-sulfur constituents such as Allicin, S-allylcysteine (SAC), diallyl disulfides (DADS), diallyl trisulfides (DATS), and methyl thiosulfonate (Banerjee et al., 2002; Qidwai and Ashfaq, 2013).

Many hypertensive patients use garlic to lower their blood pressure (Qidwai and Ashfaq, 2013). The reported effects vary from significant reduction in mean arterial pressure, drop in either SBP or DBP only, to no alteration in blood pressure at all (Banerjee et al., 2002; Mousa and Mousa, 2007; Frishman et al., 2009; Yang et al., 2011; Augusti et al., 2012). However, most studies confirm the induction of hypotensive effects by garlic and its constituents. Banerjee and Maulik reviewed pertinent literature and concluded that different forms of garlic can reduce SBP, DBP, or both (Banerjee et al., 2002). Also, an investigation by Qidwai and Ashfaq indicated that an almost 80% efficacy in anti-hypertensive effects of garlic was reported (Qidwai and Ashfaq, 2013). Interestingly, evidence from meta-analysis studies indicates that aged garlic extract (AGE) produces consistent lowering of blood pressure compared to other forms of garlic. A recent meta-analysis of randomized, controlled trials concluded that garlic supplements induce a significant reduction in both SBP and DBP by 3.75 and 3.39 mmHg, respectively (Wang et al., 2015). Similarly, another meta-analysis on randomized, controlled trials also reported a significant decrease in SBP by 4.6 ± 2.8 mmHg compared to placebo (Ried et al., 2008). Moreover, in a double-blind, parallel randomized placebo-controlled study, individuals with uncontrolled hypertension (≥140 mmHg) who were treated with 960 mg/day of AGEs for 12 weeks exhibited an average decrease of 10.2 ± 4.3 mmHg in SBP (Ried et al., 2010). This effect appears to be due to the principle constituent S-allylcysteine, which is relatively more stable in relation to allicin (Ried et al., 2010). Furthermore, in another randomized, parallel, placebo-controlled trial, patients with stage 1 hypertension ingested garlic tablets (300–1500 mg/day) for 24 weeks (Ashraf et al., 2013). This study reported a significant decrease in SBP and DBP by a maximum of 9.2 and 6.27 mmHg, respectively (Ashraf et al., 2013).

Analysis of assays on antioxidant ability of different forms of garlic demonstrate far greater potency in aged extracts than other types of garlic clove derivatives (Mathew and Biju, 2008). Indeed, AGE potently scavenges ROS (Drobiova et al., 2011; Morihara et al., 2011) leading to an increase in cellular antioxidants (Banerjee et al., 2002; Drobiova et al., 2011). In comparable studies, Drobiova et al. (2011) treated two-kidney, one-clip (2K-1C) hypertensive rats for 3 weeks with an aqueous extract of garlic (500 mg/ml), which raised the antioxidant levels by over 60% and resulted in reduction of SBP by 50%. The superoxide scavenging abilities of AGE have also been demonstrated in human neutrophils (Morihara et al., 2011). In addition, a daily 150 or 400 mg/kg dose of garlic extracts has also been reported to decrease NADPH oxidase in fructose-fed rats' aorta (Vazquez-Prieto et al., 2011).

The endogenous signaling gases, NO and H_2_S, are recognized as mediators of garlic's antihypertensive properties (Banerjee et al., 2002; Mousa and Mousa, 2007; Ried et al., 2013). In a clinical trial performed by Mousa and Mousa (2007) on stage 1 hypertensive subjects (≥140 mmHg), who consumed a daily dose of 2600 mg of garlic (one tablet comprising of 650 mg of garlic bulb—*Allium sativum*—powder) for a period of 10 days, the authors reported a significant reduction of 17 mmHg in SBP, but the DBP remained unchanged (Mousa and Mousa, 2007; **Table 6**). In mechanistic support of the blood pressure measurements, the group exposed human umbilical vein endothelial cells (HUVECs) to a garlic extract, which caused an increase in the bioavailability of NO, a potent vasodilator, by 200% (Mousa and Mousa, 2007). This is thought to occur through the reaction between NO and the sulfide components of garlic (Ku et al., 2002). An ethanolic extract of garlic (0.8 mg/ml) caused relaxation in rat pulmonary arteries pre-contracted with phenylephrine (Ku et al., 2002). Another study has demonstrated that extracts (150 and 400 mg/kg daily) of garlic not only upregulate eNOS, but also induce an increase in eNOS activity in fructose-fed rats (Vazquez-Prieto et al., 2011). In addition, garlic does not merely increase H_2_S production, but induces its synthesis for vasorelaxant activity (Benavides et al., 2007). In their study, Benavides et al. (2007) demonstrated that red blood cells synthesize H_2_S from polysulfides that were extracted from garlic. They also reported that garlic (500 μg/ml) and garlic compounds-mediated increase in H_2_S is correlated with an increase in vasorelaxant activities in rat aortic rings (Benavides et al., 2007). Moreover, treatment with 50 μM garlic-derived DADS induce an increase in expression of Connexin-43 (Cx43), a gap junction protein whose expression is correlated with a reduced VSMC proliferation and DNA synthesis (Joshi et al., 2012; **Table 4**).

Garlic's ability to inhibit ACE activity has also been recognized (Sendl et al., 1992), and in this regard gamma-glutamyl-cysteines have been identified as the antagonists (Sendl et al., 1992). In addition, a daily dose of 150 and 400 mg/kg of aqueous garlic extracts caused a reduction of VCAM-1 in fructose-fed rats (Vazquez-Prieto et al., 2011). Constituents of Garlic dampen Ang II-induced vasoconstrictor responses, antagonize endothelin-1 induced vasoconstriction, inhibit VSMCs proliferation in smooth muscles isolated from SHR and abrogate the activation of NF-κB (Banerjee et al., 2002; Castro et al., 2010; Pan et al., 2012). These effects are modulated by Allicin after the reaction of Alliin with the enzyme Alliinase (Frishman et al., 2009; Qidwai and Ashfaq, 2013).

Despite having these multifarious therapeutic effects, garlic also produces a few minor side effects. Several articles report garlic's ability to cause abdominal swelling, heartburn, flatulence, and acid reflux (Yang et al., 2011; Ried et al., 2013). Individuals under anticoagulant management are advised to avoid garlic consumption throughout the duration of treatment as the anti-hemostatic effect may be far more potent and detrimental (Qidwai and Ashfaq, 2013).

### *Andrographis paniculata* (king of bitter)

*Andrographis paniculata* is a plant that is commonly known as the “King of bitter” (Awang et al., 2012). *A. paniculata* has been part of eastern and southeastern Asian traditional medicine as a treatment for cold, CVDs (Awang et al., 2012) and fever (Kunwar et al., 2010). It has been shown to possess anti-bacterial, anti-inflammatory (Awang et al., 2012), and antioxidant effects. Several hypotensive labdane-type diterpenoid compounds have been identified in *Andrographis paniculatia* extracts. These include andrographolide, 14-deoxy-11,12-didehydroandrographolide and 14-deoxyandrographolide (Awang et al., 2012). However, no clinical trials have yet been conducted using *A. paniculata*.

Treatments with extracts of *A. paniculata* decrease ACE and ROS activities in spontaneously hypertensive rats (SHR) leading to a decrease in BP (Zhang and Tan, 1996; Table 1). Both 14-deoxy-11,12-didehydroandrographolide and 14-deoxyandrographolide (1 mg/ml in 40% ethanol, dose used: 0.1 mg for each substance) reduce vascular resistance reflected by decreased coronary perfusion pressure (an index of vascular tone) in rat isolated hearts (Langendorff model; Awang et al., 2012; Table 2). Moreover, crude extracts with high content of 14-deoxy-11,12-didehydroandrographolide induced dramatic hypotensive effects (Awang et al., 2012). This was apparently due to increased NO release which consequently induced vasodilation (Awang et al., 2012). In addition, 14-deoxy-11,12-didehydroandrographolide inhibited the rise in intracellular Ca^2+^ via receptor- and voltage-gated Ca^2+^ channels (Awang et al., 2012).

In addition to its antioxidant (Lobo et al., 2010) and anti-inflammatory (Kunwar et al., 2010) characteristics, this herb can potently inhibit the activation of NFκB (Das et al., 2012; Table 3). Other reports also indicate that *A. paniculata* exhibits anti-inflammatory activities in natriuretic peptide receptor-A (Npr1)-gene knockout mice (Das et al., 2012). Indeed, a daily 4 mg/kg dose of andrographolide caused a significant reduction in the production of NF-κB (Das et al., 2012).

**Table 3:** **Commonly used antihypertensive plants with anti-inflammatory activity**.

**Herb**	**Effect**	**Concentration/Dose**	**Experimental setting/Model**	**References**
*Allium sativum*	Inhibits NF-κB	250 mg/kg	High fructose-fed rats	Padiya et al., 2014
	Reduces VCAM-1	150 mg/kg	Fructose-fed Wistar rats	Vazquez-Prieto et al., 2011
*Andrographis paniculata*	Inhibits NF-κB	4 mg/kg	Npr1 gene-knockout mice	Das et al., 2012
*Bidens pilosa L*.	Inhibits NF-κB and TNF-alpha activation	10–20 μg/ml	LPS-stimulated RAW 264.7	Xagorari et al., 2001
		1 μM		Chiang et al., 2005
*Camellia sinensis*	Inhibits NF-κB	5–30 μM (of EGCG)	Human endothelial cells	Hong et al., 2007
	Reduces VCAM-1	10–100 μM (of EGCG)	*In vitro* endothelial cells	Ludwig et al., 2004
	Decreases TNF-α	379 mg	Obese, hypertensive humans	Bogdanski et al., 2012
*Coptis chinensis*	Decreases NF-κB	150 mg/kg	Atherosclerotic renovascular rats	Wan et al., 2013
		25 μM (of Berberine)	Rat aortic endothelial cells	Wang et al., 2009
	Inhibits VCAM-1	25 μM (of Berberine)	Rat aortic endothelial cells	Wang et al., 2009
*Coriandrum sativum*	Decreases NF-κB	150 μg/ml	LPS-stimulated RAW 264.7	Wu et al., 2010
*Crataegus* spp.	Decreases TNF-α	100 mg/kg	STZ-induced diabetic rats	Topal et al., 2013
	Decreases IL-6	100 mg/kg	STZ-induced diabetic rats	Topal et al., 2013
*Crocus sativus*	Inhibits NF-κB	0.1–0.5 mL/kg/day	Ischemia-reperfusion injury (IRI) in rats	Bharti et al., 2012
*Panax*	Inhibits NF-κB	2–5 μM (one of its components)	Mouse cardiomyocytes	Ma et al., 2014
		10 μM (one of its components)	Mouse macrophages	Wang et al., 2014
	Decreases TNF-α	10 μM (one of its components)	Mouse macrophages	Wang et al., 2014
	Decreases IL-6	10 μM (one of its components)	Mouse macrophages	Wang et al., 2014
*Salviae miltiorrhizae*	Decreases TNF-α	100 μg/ml	Human umbilical vein endothelial cells	Cho et al., 2013
	Inhibits NF-κB	100 μg/ml	Human umbilical vein endothelial cells	Cho et al., 2013
	Inhibits VCAM-1	100 μg/ml	Human umbilical vein endothelial cells	Cho et al., 2013

### *Apium graveolens* (celery)

The hypotensive effect of celery has been reported in *in vivo* animal studies. Seed extracts [300 mg/kg body weight, aqueous-ethanolic (20/80, v/v), hexanic and methanolic] of *Apium graveolens* reduce blood pressure in deoxycorticosterone acetate-induced hypertensive rats (Moghadam et al., 2013). Hexanic extract was by far the more potent in lowering BP in comparison to other solvent extracts. This was explained by greater retention of n-butylphthalide, which has been identified as the source of celery's flavor and aromatic odor (Moghadam et al., 2013). Similarly, this phthalide was reported to decrease BP in another animal model of hypertension (SHRs; Tsi and Tan, 1997).

Apigenin, a flavone isolate of *A. graveolens*, blocked aortic ring contractions caused by cumulative calcium increases in high potassium (60 mM) Krebs' solution; this was suggested to result from blocking of Ca^2+^ influx via calcium channels (voltage and receptor gated; Ko et al., 1991). The inhibition of calcium entry from extracellular sources was also responsible for apigenin-dependent relaxation of noradrenaline-preconstricted rat isolated aorta. This action was not influenced by either methylene blue, endothelial denudation, indomethacin, nifedipine, or caffeine, and the concentration of intracellular signaling molecules (cAMP, cGMP, and inositol monophosphate) were unaltered (Ko et al., 1991). Importantly, extracts and constituents of celery have been reported to lower arterial pressure in humans, possibly by lowering levels of circulating catecholamines and decreasing vascular resistance (Houston, 2005). Interestingly, this herb can reduce oxidative stress by virtue of its flavonoid content that potentiates antioxidant mechanisms (Popovic et al., 2006).

### *Bidens pilosa L*. (beggar's tick, black-jack, etc.)

This plant belongs to the family Asteraceae and has several common names: beggar's tick, black-jack, and broom stick. In addition to exhibiting antihypertensive effects, *B. pilosa* also possesses anti-cancer, anti-bacterial, anti-malarial, and anti-obesity properties (Bartolome et al., 2013). As of yet, no clinical trials have determined the potential effect of this plant on hypertension. However, extracts of its leaves were able to prevent and attenuate high blood pressure in different hypertensive rat models (SHRs and fructose-fed hypertensive rats) as well as normotensive Wistar rats (Dimo et al., 2002; Bartolome et al., 2013; Tables 2, 3). In fructose-fed rats, after 6 h of treatment with 75 and 150 mg/kg of methanolic leaf extract of *B. pilosa*, SBP was reduced by 17 and 21%, respectively (Dimo et al., 2002). Interestingly, the extract also showed preventive effect on SBP by 9 and 11% at 75 and 150 mg/kg, respectively. Using the same animal model, it was also shown that a 3-week treatment with aqueous and methylene chloride extracts of *B. pilosa* can prevent fructose-induced hypertension (Dimo et al., 2001).

There are conflicting reports regarding *B. piloas*'s effect on insulin sensitivity, with some studies showing improvement in insulin sensitivity (Dimo et al., 2002; Bartolome et al., 2013), while others reporting no effect on plasma insulin concentration (Dimo et al., 2001). However, there is a definite agreement on its vasorelaxant responses (Dimo et al., 2002; Bartolome et al., 2013). Cumulative doses of a neutral extract of *B. pilosa* (with an optimum concentration of 0.32 mg/ml) relaxed potassium chloride and noradrenaline pre-constricted rat aortas (Nguelefack et al., 2005). The mechanism of vasodilation has not been completely deciphered. It appears to be independent of ATP-sensitive potassium channels, but possibly involves a calcium channel antagonism and cyclooxygenase metabolite (Nguelefack et al., 2005). Importantly, the crude extract, its fractions or the isolated phytochemicals of *B. pilosa* have been reported to display radical scavenging ability (Bartolome et al., 2013). Two of these compounds, luteolin (a flavonoid) and ethyl caffeate (ester of hydroxycinnamic acid), exhibit potent anti-inflammatory activities. For example, luteolin blocked the effects of inflammatory cytokines, TNF-α, and IL-6 (Xagorari et al., 2001). Moreover, both luteolin (Xagorari et al., 2001) and ethyl caffeate (Chiang et al., 2005) can inhibit the pro-inflammatory transcription factor, NF-κB.

### *Camellia sinensis* (tea)

Collectively, the teas prepared from *Camellia sinensis* are the most frequently consumed beverages and are second only to water, worldwide (Faria et al., 2012). Tea has pleiotropic effects including antibacterial, anti-inflammatory (Deka and Vita, 2011), anti-cancer, anti-diabetic, as well as antihypertensive actions (Deka and Vita, 2011; Table 1). The data on the hypotensive effect of tea are not concrete. Hodgson et al. reported increases in BP after 30 min of consuming tea, with the BP returning to its baseline value after 60 min (Hodgson et al., 1999). Another study suggested that green tea causes a decrease in SBP for participants with BP of 140 mmHg or higher (Nagao et al., 2007). A meta-analysis of five trials came to conclusion of no change in blood pressure subsequent to drinking tea (Taubert et al., 2007). However, a more recent meta-analysis study of randomized controlled trials demonstrated that green tea reduces both SBP and DBP by 1.98 and 1.92, respectively (Peng et al., 2014). Likewise, other meta-analysis of randomized controlled trials came to the same conclusion that green tea reduces SBP and DBP by 1.8 and 1.4 mmHg, respectively. Interestingly, it was reported that green tea evoked a more powerful hypotensive effect compared to black tea, and that long-term tea consumption produced a more significant SBP and DBP reduction. Moreover, in a double-blind, placebo-controlled trial, obese hypertensive patients who received 379 mg green tea extract for 12 weeks exhibited a significant decrease in SBP and DBP by 4 mmHg each (Bogdanski et al., 2012). Another randomized double-blind, placebo-controlled trial concluded that hypertensive subjects who consumed 4479 mg (3 cups/day, 1493 mg each) of black tea for 24 weeks exhibited a significant reduction in both SBP and DBP by 2 and 2.1 mmHg, respectively (Hodgson et al., 2012). It is important to note here that ingestion of green or black tea (7.6 g in 400 ml water) by mild hypertensive subjects did not decrease blood pressure. On the contrary, green or black tea ingestion caused a non-significant increase in SBP/DBP by 1.7/0.9 and 0.7/0.7 mmHg, respectively (Hodgson et al., 1999).

Catechins, the major flavonoids in tea, include (−)-epicatechin (EC), (−)-epicatechin-3-gallate (ECG), (−)-epigallocatechin (EGC), and (−)-epigallocatechin-3-gallate (EGCG; Deka and Vita, 2011). EGCG constitutes the primary component of tea's total catechins (Babu and Liu, 2008; Faria et al., 2012; Slevin et al., 2012; Thomson et al., 2012). Constituents of tea lessen the risk of hypertension through several mechanisms, such as attenuation of oxidative stress (Table 1). Green tea has been reported to increase CAT antioxidant enzyme while simultaneously blocking AT_1_ receptors in streptozotocin-treated rats (Thomson et al., 2012). It has also been reported to upregulate the expression of antioxidants genes such as SOD1 and GST in C57BL/6 mice (Newsome et al., 2014). Another mechanism for oxidative stress reduction by tea is the inhibition of eNOS uncoupling (Faria et al., 2012). In addition, green tea has the capacity to scavenge superoxides (Nakagawa and Yokozawa, 2002) *in vitro* as well as attenuate NAPDH oxidase production (Ribaldo et al., 2009) in diabetic SHRs.

Flavonoids are noted for their vasorelaxant responses, including flow-mediated (Ras et al., 2011), and endothelial-dependent dilation (Oyama et al., 2010; Table 2). Black tea catechins are converted by an enzymatic (polyphenol oxidase and peroxidase) oxidative polymerization reaction to tannins: theaflavins (benztropolone ring) and thearubigins, both of which are orange-red colored polyphenolic pigments that are also potent vasodilators (Yang et al., 2011). A couple of clinical studies have reported black tea's positive effect on flow-mediated dilation (FMD), an index of endothelial function (Duffy et al., 2001; Hodgson et al., 2002). In subjects with moderately elevated cholesterol and/or triglyceride levels, consumption of black tea increased FMD in brachial arteries (Hodgson et al., 2002) as well as in patients with coronary arterial disease (450 mL of tea or water—short-term, after 2 h; and 900 mL of tea or water/day for 4 weeks long-term, *n* = 50 subjects; Duffy et al., 2001). In a clinical trial, Oyama et al. (2010) used venous occlusion strain-gauge plethysmography to demonstrate the beneficial effects of green tea catechins (580 mg/day for 4 weeks) on blood flow in forearms of smokers. The data revealed a significant increase in blood flow due to an augmented release of NO with a concomitant decrease in levels of both asymmetrical dimethylarginine (an endogenous inhibitor of eNOS) and oxidative stress (Oyama et al., 2010; **Table 6**).

The bioactive components of tea are reported to express anti-inflammatory properties (Table 3), reflected by mitigated expression and release of different cytokines. In a clinical double-blind, placebo-controlled study on 56 obese, hypertensive males and females, green tea extract (379 mg/day for 3 months) caused a decrease in BP as well as TNF-α levels (Bogdanski et al., 2012). In other studies, EGCG (Ludwig et al., 2004) and theaflavin (Lü et al., 2005) derived from tea have been reported to reduce VCAM-1 levels. In addition, EGCG was able to inhibit NF-κB activation in human endothelial cells (Hong et al., 2007). Interestingly, EGCG derived from green tea also elicited a concentration-dependent inhibition of proliferation in human aortic VSMCs by increasing HO-1 enzyme expression (Liu et al., 2014).

### *Coptis chinensis* (goldthread)

*Coptis chinensis*, commonly known as Chinese goldthread, is widely used in Chinese folk medicine (Affuso et al., 2010). Evidence indicates that goldthread, and its main component Berberine (BBR), have the ability to lower blood pressure (Affuso et al., 2010; Xiong et al., 2013). Indeed, a recent meta-analysis of twenty-seven randomized controlled trials involving 2569 patients reported that BBR can cause a significant hypotensive effect (Lan et al., 2015). In addition, this meta-analysis concluded that combined with an oral hypotensor, BBR can significantly reduce BP more than the hypotensor alone can do. The magnitude of the decrease was determined to be an average of 4.91 and 2 mmHg for SBP and DBP, respectively (Lan et al., 2015).

Several mechanisms have been proposed for Chinese goldthread's antihypertensive effect. One mechanism appears to be via amelioration of oxidative stress (Zhang et al., 2011; Wan et al., 2013; Table 1). Indeed, BBR (150 mg/kg) is reported to scavenge ROS, inhibit NADPH oxidase (Wan et al., 2013), and increase the antioxidant enzyme, SOD, in rats with atherosclerotic renovascular disease.

Constituents of goldthread also act by relaxing arterial tissues through endothelial-dependent and independent routes (Affuso et al., 2010). In chronic intermittent hypobaric hypoxic and normoxic animal models, goldthread has been shown to relax NE-induced contractions in rat isolated thoracic aortic rings (Zhang et al., 2011). The same authors also reported BBR's vasorelaxant activity on KCl-induced contractions using the same models (Zhang et al., 2011). Apparently, BBR elevates the expression of eNOS with a concomitant rise in NO release that leads to enhanced flow-mediated vasodilation (Affuso et al., 2010; Zhang et al., 2011). This dilation is likely mediated by the vasodilator PGI_2_ as well as the opening of K_ATP_ channels and blockage of Ca^2+^ influx (Zhang et al., 2011). In a clinical study, BBR (1.2 g/day for 1 month) decreased the formation of endothelial microparticles (EMPs) which are known to induce endothelial dysfunction and pro-coagulant activity in healthy humans (Wang et al., 2009; Affuso et al., 2010).

In addition, BBR isolate of Chinese goldthread inhibits endothelial injury (Wang et al., 2009) modulates inflammatory pathways through suppression of transcription factor NF-κB, VCAM-1 expression, VSMC proliferation (Affuso et al., 2010; Wan et al., 2013; Table 4). It also improves lipid profile by reducing total and LDL cholesterol, and cardiac muscle hypertrophy (Zhang et al., 2011).

**Table 4:** **Commonly used antihypertensive plants with anti-proliferative activity**.

**Herb**	**Effect**	**Concentration/Dose**	**Experimental setting/Model**	**References**
*Allium sativum*	Induces Cx43 expression	50 μM	Sprague-Dawley rat thoracic aortic VSMCs	Joshi et al., 2012
	Inhibits Ang-II-induced cell cycle progression	100 μM (two of its components)	VSMCs isolated from SHR	Castro et al., 2010
*Camellia sinensis*	Increases HO-1 enzyme	0–50 μM	Human aortic smooth muscle cells	Liu et al., 2014
*Coptis chinensis*	Inhibits cardiac hypertrophy	300 mg/kg	Rat isolated cardiomyocytes (insulin-induced hypertrophy)	Zhang et al., 2011
*Panax*	Inhibits ERK pathway activation	10% of plasma isolated from rats injected with 200 mg/kg of the extract	PDGF-treated rat VSMCs	Zhang et al., 2012
	Decreases CDK4, pRb, and cyclin D1	20–40 mg/ml	SHR thoracic aortic VSMCs	Tao and Lei, 2012
	Decreases β-galactosidase	20–40 mg/ml	SHR and WKY rat thoracic aortic VSMCs	Tao and Lei, 2012
*Salviae miltiorrhizae*	Inhibits PDGF proliferation	100 μg/ml	Sprague-Dawley rat thoracic aortic VSMCs	Cho et al., 2013

### *Coriandrum sativum* (cilantro or coriander)

In several countries, coriander (also known as cilantro or dhania) is not only used as a culinary ingredient (Anilakumar et al., 2010; Wu et al., 2010) but also as a traditional medicine for the treatment of cardiovascular and gastrointestinal diseases (Jabeen et al., 2009).

To the best of our knowledge, no clinical trials have been conducted to assess coriander's effects on BP. However, coriander has been reported to exhibit antioxidant properties (Sreelatha et al., 2009; Cioanca et al., 2013; Ramkissoon et al., 2013). In isoproterenol-induced myocardial infarction (MI) model of cardiotoxicity, coriander extract (200 and 300 mg/kg) inactivated β-adrenoceptor-induced ROS production and also prevented MI by inhibiting myofibrillar damage (Patel et al., 2012; Table 1). Coriander leaves' extracts also increased antioxidants enzymes (Sreelatha et al., 2009) and its seeds' powder (5 and 10%) showed similar effect on the antioxidant GPX (Anilakumar et al., 2010). Other studies have also reported similar antioxidant activities of coriander (Cioanca et al., 2013; Ramkissoon et al., 2013).

Vasodilatory effects of coriander are well-established. Indeed, intravenous application of aqueous methanolic extract of dried, ground coriander seeds (1–30 mg/ml) produced a dose-dependent fall in SBP, DBP, and mean arterial blood pressure (MABP) in normotensive Sprague-Dawley rats by 40.84 ± 6.34% (Jabeen et al., 2009). The same report also showed that coriander fruit extracts produced dose-dependent relaxation of pre-constricted (phenylephrine and potassium chloride) rabbit aortas, and this response was atropine and calcium-channel dependent (Jabeen et al., 2009). Further, the same extracts showed diuretic affects as well. The active component of which should act synergistically with the vasoactive constituent to complement the treatment and management of hypertension (Jabeen et al., 2009). Moreover, coriander acts as an inhibitory agent to reduce the activities of NF-κB and iNOS (Wu et al., 2010).

### *Crataegus* spp. (hawthorns)

Hawthorns (hawberry or thorn apple) plants are shrubs that belong to a genus comprising almost 300 species (Tassell et al., 2010) that have been used in traditional medicine for thousands of years (Tassell et al., 2010; Asher et al., 2012). Hawthorns have been used for treatment of CVDs since the seventeenth century (Asher et al., 2012).

Modest decreases in blood pressure have been observed in a few human-based studies with a demographic of hypertensive patients (Walker et al., 2002; Tassell et al., 2010). In a randomized, double-blind, placebo-controlled study where mildly hypertensive subjects were treated with 500 mg of hawthorn extract for 10 weeks, a promising tendency for a reduction in DBP (by 13.1 mmHg) was reported (Walker et al., 2002). It is argued that the dose and duration were not sufficient for a more effective result to be noted. Indeed, in phytotherapy practice, a significant decrease in BP is only noted after a longer duration and higher doses (Bone and Mills, 2013), In another randomized, double-blind, placebo-controlled clinical study, administration of hydro-alcoholic extracts of *Crataegus curvisepala* Lind flowers to hypertensive patients (age range 40–60 years) for 3 months induced a decrease in both SBP and DBP by around 13 and 8 mmHg, respectively (Asgary et al., 2004).

The aforemnetioned antihypertensive actions are credited to the plant's multiple components: flavonoids (hyperoside, quercetin, rutin, and vitexin) and oligomeric proanthocyanidins (OPCs, epicatechin, procyanidin, and procyanidin B-2; Valli and Giardina, 2002; Houston, 2005; Asher et al., 2012; Yang and Liu, 2012). Quercetin, a major polyphenolic flavonoid in hawthorn shrubs, expresses numerous bioactive functions including anti-oxidant, anti-inflammatory, and vasorelaxant effects. Quercetin supplement intervention studies have demonstrated a reduction in blood pressure of hypertensive subjects (Larson et al., 2012). Interestingly, hawthorn extracts have an effect on both VSMCs and endothelial cells (Tassell et al., 2010). The latter interaction is attributable to increased NOS activity and hence NO release (Brixius et al., 2006; Anselm et al., 2009), possibly due to an enhanced phosphorylation of eNOS at serine 1177 (Brixius et al., 2006; Anselm et al., 2009; Table 2). In porcine isolated coronary arterial rings, WS 1442 (an extract of Crataegus leaves with flowers) induced endothelium-dependent relaxation through the activation of multiple signaling pathways, including ROS, Src, PI_3_-kinase, Akt, and eNOS (Anselm et al., 2009). Moreover, WS 1442 caused endothelium-dependent and NO-mediated vasorelaxation of phenylephrine-preconstricted rings of rat aorta as well as human internal mammaria (Brixius et al., 2006). In the L-NAME-induced hypertension model, the blood pressure decreased after the administration of *Crataegus tanacetifolia* leaf extract or its isolate, hyperoside. Both *C. tanacetifolia* extract and hyperoside display protective effects at multiple levels. These include improving hyperlipidemia, decreasing body weight, resolving hyperplasia, reducing thickness of the vascular medial layer as well as improving kidney function. Such effects appear to be mediated by an increase in diuretic activity, efflux of water and sodium, as well as expression of NOS enzyme. Together, all of these mechanistic actions contribute to the amelioration of hypertensive outcome (Koçyildiz et al., 2006).

As part of the integrated cardiovascular beneficial bioprocesses, hawthorn has the capacity to scavenge ROS (Tassell et al., 2010; Cheng et al., 2013; Table 1), up-regulate antioxidant enzymes (SOD, CAT) and augment the concentration of the reducing glutathione (GSH; Tassell et al., 2010). Moreover, hawthorn extracts express anti-inflammatory activity, which is mirrored by the decline in concentrations of NF-κB, TNF-α (Topal et al., 2013), VCAM-1 (Shin et al., 2012), iNOS, and IL-6 (Topal et al., 2013).

### *Crocus sativus* (saffron)

Saffron (common name), a plant indigenous to Southwest Asia (Iran, Pakistan, and India), Spain, Greece, and Morocco, is a stemless herb whose medicinal values have been sought for over 4000 years (Srivastava et al., 2010). Saffron's main components include crocin, picrocrocin, safranal, and crocetin (Srivastava et al., 2010; Mehdizadeh et al., 2013) and these molecules exhibit different mechanisms of action (Mokhtari-Zaer et al., 2015).

Several reports support the use of saffron for anti-hypertensive benefits. A clinical study reported that 400 mg of saffron tablets administered for 7 days were able to significantly reduce the SBP and mean arterial pressure in healthy humans by 11 and 5 mmHg, respectively (Modaghegh et al., 2008). Saffron demonstrates vasorelaxant activities in different animal models. Extracts of *C. sativus* petals (rich in flavonoids and anthocyanins) dose-dependently reduced the BP of male Sprague-Dawley rats, possibly by modulating peripheral vascular resistance (Fatehi et al., 2003). Moreover, extract of *C. sativus* stigma (10 mg/kg), and two of its primary components [Crocin (200 mg/kg) and Safranal (1 mg/kg)], attenuated MABP in normotensive and desoxycorticosterone acetate (DOCA)-salt induced hypertensive male Wistar rats (Imenshahidi et al., 2010). More recently, it was shown that chronic administration of safranal (1, 2, and 4 mg/Kg/day) reduced SBP in DOCA-salt hypertensive but not normotensive rats (Imenshahidi et al., 2015).

Saffron relaxes non-vascular muscles as well. Indeed, extracts of saffron decreased contractility and heart rate of guinea-pig isolated perfused hearts (Langendorff procedure) by blocking Ca^2+^ channels, opening potassium channels, and antagonizing β-adrenoceptors (Boskabady et al., 2008; Table 2). In addition, safranal (0.1–0.5 mL/kg daily) offers protection in a rat model of myocardial ischemia-reperfusion injury via enhanced phosphorylation of protein kinase B (Akt)/glycogen synthase kinase-3β (GSK-3β)/eNOS pathway, attenuation of IKK-β/NF-κB activity, normalization of the antioxidant reserve and up-regulation of the anti-apoptotic route (Bharti et al., 2012; Table 2).

Saffron's antioxidant ability has also been widely reported. It was shown to reduce oxidative stress (El-Beshbishy et al., 2012; Mehdizadeh et al., 2013) and increase antioxidant enzymes, such as SOD (Premkumar et al., 2003; El-Beshbishy et al., 2012), CAT, GPX, and reduced GSH (Premkumar et al., 2003;Table 1). 20–80 mg/kg of saffron's aqueous extracts were able to increase antioxidants levels in genotoxin-treated mice (Premkumar et al., 2003). Crocin treatment (200 mg/kg for 7 days) of beryllium chloride-induced model of oxidative stress in male Wistar rats resulted in a significant decline in oxidative stress and the corresponding up-regulation of antioxidant enzymes (El-Beshbishy et al., 2012). Moreover, saffron and its constituents also possess an inherent ability to inhibit inflammatory pathways, including NF-κB (Nam et al., 2010; Bharti et al., 2012) and TNF-α expression (Nam et al., 2010).

### *Cymbopogon citratus* (lemongrass)

Lemongrass (common name) has been widely used in traditional medicine of Brazil, China, and Southern Asia (Devi et al., 2012). It has been reported to possess antihypertensive properties, which have been attributed to its active phytochemicals, the principle of which being Citral (Devi et al., 2011, 2012). However, no clinical trials have yet investigated the effect of Lemongrass on BP.

The relaxant effect of lemongrass has been demonstrated in several different tissues, including the rabbit ileum (Devi et al., 2011), rat aortic rings (Devi et al., 2012), and the rat mesentery (Bastos et al., 2010; Table 2). For instance, Citral or crude extracts of *C. citratus* (leaves, stems, or roots) generated a dose-dependent vasorelaxation in phenylephrine pre-constricted aortic rings from male WKRs or SHRs (Devi et al., 2012). Further, the underlying mechanism for this relaxation appeared to be mediated by activation of NO and/or the inhibition of calcium channels (Devi et al., 2012). Likewise, administration of an intravenous bolus of Citronellol, an acyclic monoterpenoid isolated from lemongrass, to male Wistar rats produced a hypotensive response. This hypotensive effect was not affected by L-NAME, indomethacin, atropine, or hexamethonium (Bastos et al., 2010). Citronellol also induced relaxation of rat superior mesenteric artery via an endothelium-independent mechanism. Moreover, arteries denuded of endothelium were not reliant on tetraethylammonium-dependent potassium channels. Rather, citronellol acted by inhibiting Ca^2+^-influx through voltage operated calcium channels (VOCCs) as well as regulating IP_3_- and caffeine-gated intracellular Ca^2+^ stores (Bastos et al., 2010).

Lemongrass is known to display moderate antioxidant activity. In male rats treated with H_2_O_2_, 100 mg/kg of lemongrass was able to reduce oxidative stress and increase GSH expression in testes (Rahim et al., 2013). Results from another study showed an increase in antioxidant enzymes and molecules such as SOD and GSH in murine lungs after administering 5 and 10 μg of lemongrass' extracts (Tiwari et al., 2010). Lemongrass was reported to suppress ROS molecular activity (Tiwari et al., 2010; Koh et al., 2012). In addition, lemongrass' Citral contributes to the anti-inflammatory pathways by inhibiting NF-κB (Lee et al., 2008; Francisco et al., 2013) and iNOS activity (Lee et al., 2008; Figueirinha et al., 2010).

### *Hibiscus sabdariffa* (roselle)

Hibiscus, widely known as roselle, is used for hypertension, fever, and other diseases in folk medicine. Different parts of this plant (buds, calyx, flowers, leaves, and petals—fresh or dried) are used for health purposes and as refreshing beverages, food items (jams, preserves), or lotions.

Roselle's blood pressure lowering effects have been extensively reported in both animal (Odigie et al., 2003; Ali et al., 2005; Ajay et al., 2007; Mojiminiyi et al., 2007; McKay et al., 2010; Ojeda et al., 2010; Inuwa et al., 2012; Hopkins et al., 2013) and human studies (Onyenekwe et al., 1999; Herrera-Arellano et al., 2004, 2007; Mojiminiyi et al., 2007; Mozaffari-Khosravi et al., 2009; Inuwa et al., 2012; Hopkins et al., 2013). In a randomized, double-blind, Lisinopril-controlled clinical trial, antihypertensive effects were notable subsequent to treatment with dried extract of calyx (250 mg) for 4 weeks in patients with stage 1 or 2 hypertension (Herrera-Arellano et al., 2007; **Table 6**). Indeed, a drop of BP from 146.48/97.77–129.89/85.96 mmHg was noticed (Herrera-Arellano et al., 2007). In yet another randomized controlled trial, hypertensive patients ingested 10 g/day of Roselle's calyx. After 4 weeks, a significant decrease in SBP and DBP by 15.32 and 11.29 mmHg, respectively was reported (Herrera-Arellano et al., 2004). Additional support for hibiscus' therapeutic role in ameliorating hypertension is provided by a report which shows that in mild and pre-hypertensive patients (65 subjects—30–70 years old), consuming roselle's tea (240 ml—three times a day for 6 weeks) significantly reduces SBP, DBP, and MAP by 7.2, 3.1, and 4.5 mmHg, respectively (McKay et al., 2010).

Different mechanisms for roselle's antihypertensive effect have been reported. Roselle primes the vasorelaxant pathways of both endothelial cells (Ajay et al., 2007; Herrera-Arellano et al., 2007) and VSMCs (Ali et al., 2005; Ajay et al., 2007). Its relaxant effect is mediated through an increased production of NO (Ajay et al., 2007; Alarcon-Alonso et al., 2012), inhibition of Ca^2+^ channels (Ajay et al., 2007) and opening of K_ATP_ channels (Sarr et al., 2009). Additionally, it has been shown to inhibit cardiac hypertrophy (Odigie et al., 2003; Inuwa et al., 2012), and decrease heart rates in rats (Odigie et al., 2003).

A strong body of evidence is reported to support roselle's ability as a diuretic agent (Onyenekwe et al., 1999; Ali et al., 2005; Herrera-Arellano et al., 2007; Alarcon-Alonso et al., 2012). One clinical study detected a lower concentration of uric acid in urine of healthy humans consuming roselle (Ali et al., 2005). Another clinical study reflected the decrease in blood sodium content of stage 1 and 2 hypertensive humans after 4 weeks administration of 250 mg of roselle's extracts (Herrera-Arellano et al., 2007), thus verifying its multiple diuretic properties. This diuretic activity is related to the vasorelaxant effect, as NO elevation is positively correlated with increases in renal filtration rates (Alarcon-Alonso et al., 2012).

Roselle exhibits potent antioxidant function (Ali et al., 2005; McKay et al., 2010). Its anthocyanin extracts (2 mg/ml) were reported to reduce oxidative stress, potentially by scavenging free radicals in livers of CCl_4_-treated rats (Ajiboye et al., 2011). In a clinical study, roselle's aqueous extracts enhanced the concentration of cellular antioxidants in healthy humans (Frank et al., 2012; Table 1). Moreover, it blocks the oxidation of LDL, indicating its role as an anti-atherogenic (Lin et al., 2011). Roselle also shows anti-inflammatory capacity by inhibiting not only ACE activity (Herrera-Arellano et al., 2007; Ojeda et al., 2010) but also the proliferation of VSMCs (Lin et al., 2011). The effect of roselle on ACE activity was confirmed in a Lisinopril-controlled clinical trial, where 250 mg of its extract was administered to patients with stage 1 or 2 hypertension (Herrera-Arellano et al., 2007).

### *Nigella sativa* (black cumin; seed of blessing)

Black cumin, also known as Habbatul barakah (seed of blessing), has been used in the kitchens of Europe, the Middle East, Africa, and South and Southwest Asia for centuries (Ahmad et al., 2013; Leong et al., 2013; Tables 2, 5). In addition to its antihypertensive role, black cumin is also effective against diabetes and gastrointestinal diseases (Leong et al., 2013). Thymoquinone (TQ), one of the most abundant and bioactive components in *Nigella sativa*'s seeds, has been identified as the major element in its healing effects (Ahmad et al., 2013).

**Table 5:** **Commonly used antihypertensive plants with diuretic activity**.

**Herb**	**Effect**	**Concentration/Dose**	**Experimental setting/Model**	**References**
*Hibiscus sabdariffa*	Lowers uric acid concentration	16 g/day	Healthy men	Ali et al., 2005
		1500–2500 mg/kg	SHR	Alarcon-Alonso et al., 2012
	Reduces plasma Na^+^ levels	250 mg	Stage 1 and 2 hypertensive humans	Herrera-Arellano et al., 2007
*Nigella sativa*	Increases Na^+^, K^+^, and Cl^−^ in urine	5 ml/kg/day	SHR	Zaoui et al., 2000

*N. sativa*, and its constituents, have been reported to lower blood pressure in humans and in different animal models of hypertension (Zaoui et al., 2000; Khattab and Nagi, 2007; Dehkordi and Kamkhah, 2008; Fallah Huseini et al., 2013) and also decrease heart rate (Leong et al., 2013). For example, L-NAME-induced hypertension in rats was ameliorated by concomitant treatment with thymoquinone (0.5 and 1 mg/kg/day for 4 weeks; Khattab and Nagi, 2007). Improvement in renal function and antioxidant activity were also noted, evident by the reduced serum creatinine or increased glutathione in control vs. L-NAME treated rats, respectively (Khattab and Nagi, 2007). Likewise, in SHRs, an oral dose of dichloromethane extract of black cumin seeds (0.6 ml/kg/day for 15 days) reduced mean arterial pressure by 22% and increased diuretic activity (Khattab and Nagi, 2007), mirrored by urinary excretion of urea, Cl^−^, Na^+^, and K^+^ (Zaoui et al., 2000; Table 5).

A randomized, double-blind placebo-controlled clinical trial of the effects of *N. sativa*'s seed extract administrated orally (either 100 or 200 mg, two times per 24 h for 8 weeks) to mild hypertensive male patients recorded a dose-dependent fall in both SBP and DBP, in the two treated groups compared to placebo (Dehkordi and Kamkhah, 2008; Table 6). For the 200 mg dose, there was a decrease of around 2.2 and 2 mmHg in SBP and DBP, respectively. Moreover, the *NS*'s extract reduced total cholesterol as well as low density lipoprotein (LDL)-cholesterol relative to pretreatment concentrations (Dehkordi and Kamkhah, 2008). Again, in a randomized, placebo-controlled, double-blind study with 70 healthy subjects, *N. sativa* oil caused a significant decrease of 10.6 and 9.6 mmHg, respectively in both SBP and DBP (Fallah Huseini et al., 2013).

**Table 6:** **Commonly used plants that were studied in clinical trials, and details of these trials**.

**Herb**	**Design**	**Population size**	**Condition**	**Dose**	**Duration**	**Effect**	**Magnitude of change**	**References**
*Allium sativum*	Double-blind, parallel, randomized, placebo-controlled	50	Uncontrolled hypertension	960 mg/day aged garlic extract	12 weeks	SBP decrease	10.2 ± 4.3 mmHg	Ried et al., 2010
	Placebo-controlled, crossover	6	Mild hypertension	2600 mg/day (4 tablets, 650 mg each) garlic powder	10 days	SBP decrease	17 mmHg	Mousa and Mousa, 2007
	Double-blind, parallel, randomized, placebo-controlled	79	Uncontrolled hypertension	480 mg/day aged garlic extract	12 weeks	SBP decrease	11.8 ± 5.4	Ried et al., 2013
	Randomized, parallel, placebo-controlled	210	Stage 1 hypertension	300–1500 mg/day garlic powder	24 weeks	SBP and DBP decrease	9.2 and 6.26 mmHg	Ashraf et al., 2013
*Camellia sinensis*	Double-blind, placebo-controlled	20	Mild hypertension	7.6 g tea leaves in 400 ml water	1 h	SBP and DBP increase	1.7 and 0.9 mmHg (green tea)	Hodgson et al., 1999
							0.7 mmHg each (black tea)	
	Randomized, parallel, placebo-controlled	56	Obese, hypertension	379 mg green tea extract	12 weeks	SBP and DBP decrease	4 each mmHg	Bogdanski et al., 2012
	Randomized, parallel, placebo-controlled	95	Mild hypertension	4479 mg (3 cups/day, 1493 mg each) black tea	24 weeks	SBP and DBP decrease	2 and 2.1 mmHg	Hodgson et al., 2012
*Crataegus* spp.	Randomized, double-blind, placebo-controlled	36	Mild hypertension	500 mg/day extract	10 weeks	DBP	13.1 mmHg	Walker et al., 2002
	Randomized, double-blind, placebo-controlled	92	Mild hypertension	2.7–3 mg/day flavonoids (contained in Hydro-alcoholic extract)	4 months	SBP and DBP decrease	13 and 8 mmHg	Asgary et al., 2004
*Crocus sativus*	Randomized, double-blind, placebo-controlled	30	Healthy	400 mg/day	7 days	SBP and MAP decrease	11 and 5 mmHg	Modaghegh et al., 2008
*Hibiscus sabdariffa*	Randomized, captopril-controlled	75	Mild to moderate hypertension	10 g/day dried calyx	4 weeks	SBP and DBP decrease	15.32 and 11.29 mmHg	Herrera-Arellano et al., 2004
	Randomized, double-blind, Lisinopril-controlled	193	Stage 1 and 2 hypertension	250 mg dried calyx extract	4 weeks	SBP and DBP decrease	16.59 and 11.8 mmHg	
	Randomized, double-blind, placebo-controlled	65	Pre- and mild hypertension	720 mL/day (3 servings, 240 mL each) hibiscus tea (3.75 g hibiscus)	6 weeks	SBP, DBP, and MAP decrease	7.2, 3.1, and 4.5 mmHg	McKay et al., 2010
*Nigella sativa*	Randomized, double-blind, placebo-controlled	108	Mild hypertension	200 and 400 mg/day (100 and 200 twice a day) aqueous seed extract	8 weeks	SBP and DBP decrease	2.2 and 1.1 mmHg	Dehkordi and Kamkhah, 2008
						LDL-cholestrol reduction		
	Randomized, double-blind, placebo-controlled	70	Healthy	5 mL/day (2.5 twice a day) NS oil	8 weeks	SBP and DBP decrease	10.6 and 9.6 mmHg	Fallah Huseini et al., 2013
*Panax*	Randomized, placebo-controlled	90	Mild hypertension	300 mg/day *P. ginseng* extract	8 weeks	SBP and DBP decrease	3.1 and 2.3 mmHg	Rhee et al., 2014
	Randomized, double-blind, placebo-controlled	64	Essential hypertension	3 g/day *P. quinquefolius*	12 weeks	SBP decrease	17.4 mmHg	Mucalo et al., 2013
	Randomized, double-blind, crossover	23	Healthy	400 mg	3 h	SBP and DBP decrease	4.8 and 3.6 mmHg	Jovanovski et al., 2014

Another route by which black cumin attenuates hypertension is by vasorelaxant means. This is illustrated by its ability to inhibit Ca^2+^ channels (voltage-gated and ligand-gated) leading to concomitant relaxation of rat aorta (Leong et al., 2013). Moreover, TQ is reported to inhibit the release of vasoconstrictor metabolites of COX-1 (Ahmad et al., 2013) and COX-2 (Ahmad et al., 2013; Kundu et al., 2013).

TQ's antihypertensive effects are partly due to its antioxidant activities as it lowers oxidative stress (Khattab and Nagi, 2007; Ahmad et al., 2013). Another mechanism that may explain the hypotensive effect of black cumin pertains to its diuretic action (Zaoui et al., 2000; Ahmad et al., 2013; Leong et al., 2013). This has been highlighted by the ability of black cumin seeds to increase urea, calcium, sodium, and potassium in urine of rats (Zaoui et al., 2000; Leong et al., 2013), where it also increases renal filtration and urinary output (Leong et al., 2013). Yet another mechanism that underlies black cumin's actions is its anti-inflammatory property since it inhibits the generation of TNF-α and NF-κB (Kundu et al., 2013).

### *Panax* (ginseng)

For centuries, the species *Panax*, especially the Asian variety, has been used in folk medicine (Jang et al., 2011; Kim, 2012). Ginseng is prepared and administered in various forms, either as a solid: tablets, capsules, dried roots; or as a liquid: oil, extracts or tea (Valli and Giardina, 2002). *P. ginseng* (Asian or Korean ginseng), *P. quinquefolius* (American ginseng), *P. japonicas* (Japanese ginseng) and *P. notoginseng* (Chinese ginseng) are the four most common species of *ginseng* (Valli and Giardina, 2002; Kim, 2012). Interestingly, these—as a group—have the most reported hypotensive effects. Heterogeneous triterpenoid saponins and steroid glycosides or ginsenosides (or panaxosides) are the active principle components of *ginseng* (Valli and Giardina, 2002; Kim, 2012). In addition to anti-hypertensive effects, ginseng also plays anti-carcinogenic and antidiabetic roles (Choi et al., 2013).

Although *ginseng*'s blood pressure lowering effect is widely reported (Jeon et al., 2000; Valli and Giardina, 2002; Jang et al., 2011; Kim, 2012; Mucalo et al., 2013; Tables 1, 2), conflicting reports of elevated blood pressure also exist (Valli and Giardina, 2002; Jang et al., 2011; Kim, 2012). Contextually, low doses of *ginseng* raise BP, while higher concentrations are hypotensive (Jang et al., 2011). A probable explanation for this phenomenon is the varied action of different ginsenosides (Valli and Giardina, 2002). Interestingly, Kim (2012) has used the term “normalize” to describe hypotensive and hypertensive actions of *ginseng*. Therefore, these studies suggest that *ginseng* rheostatically adjusts the BP level in hypotensive patients (Kim, 2012), possibly by tuning vascular function, modulating the autonomic nervous system, or regulating the arterial baroreflex.

Several clinical trials have been conducted to assess the efficacy of ginseng in modulating BP (Table 6). In one trial, *P. ginseng* extract rich in ginsenoside protopanaxatriol (300 mg/day) was administered to mild hypertensive patients and caused a significant decrease of 3.1 and 2.3 mmHg in SBP and DBP, respectively (Rhee et al., 2014). Furthermore, a randomized, double-blind, placebo-controlled trial where hypertensive patients ingested American ginseng (3 g/day) for 12 weeks showed that SBP was significantly lowered by 11.7% (17.4 mmHg; Mucalo et al., 2013). Along the same line, another randomized, double-blind, crossover trial reported that central SBP and DBP of healthy subjects was significantly reduced by 4.8 ± 6.8 and 3.6 ± 6.4 mmHg, respectively, after hours of ingesting Ginsenoside Rg3-enriched *P. ginseng* (400 mg; Jovanovski et al., 2014).

The primary mechanism associated with *ginseng*-induced hypotensive effect is attributed to an improvement in arterial function. Indeed, *ginseng* causes a dramatic increase in eNOS expression and NO production (Valli and Giardina, 2002; Jang et al., 2011, 2012; Hong et al., 2012; Pan et al., 2012; Table 2). Ginsenoside Rg3 (red *ginseng*) is known to activate eNOS (Valli and Giardina, 2002; Jang et al., 2011), increase NO and cGMP levels, as well as activate Ca^2+^-gated potassium channels (Kim et al., 1999b). Ginsenosides mediate vasorelaxation of different vessels in different animals: rat aortas (Kim et al., 1999a), murine coronary arteries (Pan et al., 2012), and monkey cerebral arteries (Toda et al., 2001). In the same context, this relaxation may be aided by ginseng's ability to diminish secretion of adrenal catecholamines in hypertensive rats (Jang et al., 2011).

*Ginseng* also elicits an anti-proliferative effect on VSMCs, and hence, it can be expected to possess antihypertensive and anti-atherosclerotic capacities (Table 4). Importantly, results of a clinical trial indicate that administering 3 g/day of *P. quinquefolius* for 12 weeks improves arterial stiffness in hypertensive patients (Mucalo et al., 2013; Table 6). Further support for this beneficial effect of ginseng comes from another clinical trial, where taking 400 mg of *P. ginseng* caused a significant reduction in aortic Alx, a marker of arterial stiffness (Jovanovski et al., 2014; Table 6). It has been reported that Chinese *ginseng* blocks the activation of extracellular signal-regulated protein kinases (ERK) pathway, and therefore inhibits PDGF-induced VSMCs proliferation (Zhang et al., 2012). Moreover, Chinese and other *ginsengs* reduced vascular aging of SHRs compared to WKY rats; this was reflected by inhibition of proliferation of VSMCs isolated from both WKY and SHRs. The mechanistic pathway for the effect of *ginseng* on SHR VSMCs entailed the decrease in the number of senescence-associated β-galactosidase (SA-β-gal) positive cells. In the SHR, expression of p16, and retinoblastoma protein (Rb) was elevated whereas that of cyclin D1 and cyclin-dependent kinase 4 (CDK4) was decreased (Tao and Lei, 2012). Further, red ginseng also attenuated Ang II-induced VSMC growth (Kim, 2012).

Amongst *ginseng*'s other hypotensive mechanisms is its antioxidant ability (Table 1). In this regard, it has been reported that ginsenoside Rg1 (60–120 μM) inhibits oxidative stress (Doh et al., 2013), possibly by increasing antioxidant enzymes (Zhu et al., 2009) and scavenging free radicals (Zhu et al., 2009). In addition to its antioxidant effect, *ginseng* exhibits anti-inflammatory properties. Indeed, Asian *ginseng* (red) inhibits the release of TNF-α (Kim, 2012) as well as attenuates NF-κB and p38 mitogen activated protein kinase (MAPK) pathways (Bak et al., 2012). It has been suggested that the consequence of this effect is a reduction in the vasoconstrictor activity of COX-2 enzyme (Bak et al., 2012; Table 3).

### *Salviae miltiorrhizae* (chinese sage)

*Salviae miltiorrhizae*, known as danshen or red/Chinese sage, is one of the oldest and most frequently consumed Chinese traditional herbs (Cho et al., 2013; Jiang et al., 2013a) and is commonly used for the treatment of CVDs (Ng et al., 2011; Cho et al., 2013; Jiang et al., 2013a). Danshen's most effective components include: salvianolic acid A (SalA), salvianolic acid B (SalB), danshensu, and tanshinones (Ng et al., 2011; Jiang et al., 2013a).

Danshen relaxes the vasculature via endothelium-dependent and endothelium-independent mechanisms. A combination treatment of danshen and gegen (*Pueraria lobata*) was shown to lower blood pressure in SHRs (Ng et al., 2011) and to induce relaxation of porcine coronary arteries (Hu et al., 2012), rat aorta (Ng et al., 2011), and basilar arteries (Lam et al., 2010; Table 2). Similarly, dihydrotanshinone (lipophilic constituent of danshen) is a vasorelaxant of rat coronary arteries (Lam et al., 2008). Danshen's endothelium-dependent relaxations occur via an NO-dependent mechanism (Chan et al., 2011; Ng et al., 2011; Shou et al., 2012). SalB-derived vasodilation in rabbit aorta is mediated by the NO-sGC-cGMP pathway (Shou et al., 2012). Other studies have also demonstrated that L-NAME inhibits eNOS and blocks the activity of danshen (Chan et al., 2011; Shou et al., 2012), further verifying the NO-dependent mechanism of vasodilation. In VSMCs, danshen exhibits its vasodilating effect by opening of K_ATP_, K_ir_, and K_v_ channels (Ng et al., 2011; Jiang et al., 2013a) as well as blocking the Ca^2+^ influx (Lam et al., 2008; Hu et al., 2012).

Apart from its vasodilatory capacity, danshen expresses additional anti-hypertensive parameters such as antioxidative, anti-proliferative, and anti-inflammatory activities. Its extracts have been shown to decrease ROS production in rat thoracic aorta (Cho et al., 2013). In a randomized, placebo-controlled clinical study involving chronic heart disease patients, danshen's hydrophilic extract (5 g/twice a day/60 days) increased antioxidative enzymes like CAT, SOD, and the tripeptide glutathione (Qian et al., 2012; Table 1). Danshen was also reported to inhibit PDGF-induced proliferation of VSMCs (Cho et al., 2013; Table 4). The anti-inflammatory capacity of danshen was demonstrated by virtue of its ability to inhibit TNF-α, NF-κB production, and VCAM-1 expression (Cho et al., 2013) in HUVECs (Table 3). Taken together, these results illustrate the underlying molecular mechanism for danshen's antihypertensive effect.

### *Zingiber officinale* (ginger)

Ginger, a very common culinary ingredient, is reported to possess hypotensive properties. In a clinical study, oral (70–140 mg/kg) or intravenous (1.75–3.5 mg/kg) administration of two bioactive constituents of ginger, namely (6)-gingerol and (6)-shogoal, produced triphasic blood pressure profiles: initial rapid fall, intermediate rise, and finally a delayed decrease in BP (Suekawa et al., 1984). Indeed, [6]-gingerol is now considered a novel angiotensin II type 1 receptor antagonist with an IC_50_ of 8.17 × 10^−6^ M (Liu et al., 2013).

The aqueous extract of ginger (0.05 mg/ml) has also been reported to inhibit lipid peroxidation as well as ACE in rat hearts (Akinyemi et al., 2013). In addition, zingerone, another active compound in ginger, can potently scavenge oxidant molecules like peroxynitrite (Shin et al., 2005). Recently, it was found that ginger not only reduces levels of total cholesterol, triglyceride, LDL, and vLDL, but it can also inhibit ACE-1 activity (Akinyemi et al., 2014).

## Conclusion

With CVD remaining as a leading cause of worldwide mortality, the search for more effective treatments ought to be of prime importance. An approach that appears promising is CAM (Frishman et al., 2009; Su and Li, 2011; Orekhov et al., 2013). Not surprisingly, of all small-molecule new chemical entities introduced as drugs during the last three decades, a significant fraction were either obtained from or inspired by nature (Newman and Cragg, 2012). Perhaps this could explain, at least partly, the “phenomenon” that more American patients visit CAM providers than primary care physicians (Eisenberg et al., 1998; Tachjian et al., 2010). Of relevant interest, it is important to note that herbal consumption appear to be the most common type of CAM among CVD patients (Yeh et al., 2006).

In this first part of our review, we discussed the mechanisms of action of several plants that are most commonly used in the treatment or management of hypertension.

The evidence presented is strongly indicative of the notion that herbs and plants are becoming part of evidence-based medicine in the prevention and/or treatment of CVD. The pharmacological actions of herbs or herbal isolates appear to favorably modulate several parameters implicated in the pathogenesis of blood pressure, including but not limited to ROS production, VSMC phenotype, endothelial function, platelet activation, pro-inflammatory signaling, and gene expression. With such a broad spectrum of actions, one may predict that herbal remedies will receive even more attention in the coming years, perhaps accentuating the need for further experimentations and clinical trials. Indeed, the lack of sufficient clinical trials constitutes a significant limitation on their use at the present time. Of equal importance, it may be advisable that patients be appropriately educated, particularly in relation to herbs whose consumption has been considered safe for thousands of years (black cumin, Chinese sage, coriander, garlic, ginger, ginseng, and tea), and has been supported by sound scientific evidence such as one based on clinical trials with large population groups. It is important to note that there are herbs and plants that could actually raise blood pressure and thus should be avoided by hypertensive patients. There are also other limitations for herbal therapy of hypertension. These limitations would be discussed in the second part of this review.

## Author contributions

All authors contributed to the writing. AE conceived, designed and revised the manuscript.

## Funding

This publication was made possible by grant # NPRP 4-571-3-171 from the Qatar National Research Fund (a member of Qatar Foundation). The Statements made herein are solely the responsibility of the authors. This grant was awarded to AE.

### Conflict of interest statement

The authors declare that the research was conducted in the absence of any commercial or financial relationships that could be construed as a potential conflict of interest.
